# A Self-Administered Method of Acute Pressure Block of Sciatic Nerves for Short-Term Relief of Dental Pain: A Randomized Study

**DOI:** 10.1111/pme.12338

**Published:** 2014-01-08

**Authors:** Xiaolin Wang, Wanghong Zhao, Ye Wang, Jiao Hu, Qiu Chen, Juncai Yu, Bin Wu, Rong Huang, Jie Gao, Jiman He

**Affiliations:** * Pain Medicine Program, IDD, Nanfang Hospital, Southern Medical UniversityGuangzhou, China; † Pain Medicine Program, Department of Dentistry, School of Stomatology, Nanfang Hospital, Southern Medical UniversityGuangzhou, China; ‡ Pain Medicine Program, Teaching Hospital of Chengdu, University of Traditional Chinese MedicineGuangzhou, China; § Anhui Province Hospital, Anhui Medical UniversityHefei, China; ¶ Department of Medicine, Alpert Medical School, Brown UniversityProvidence, Rhode Island, USA

**Keywords:** Pain, Sciatic Nerve, Complementary Therapies, Pressure, Self-Administered Method, Dental Diseases

## Abstract

**Objectives:**

While stimulation of the peripheral nerves increases the pain threshold, chronic pressure stimulation of the sciatic nerve is associated with sciatica. We recently found that acute pressure block of the sciatic nerve inhibits pain. Therefore, we propose that, the pain pathology-causing pressure is chronic, not acute. Here, we report a novel self-administered method: acute pressure block of the sciatic nerves is applied by the patients themselves for short-term relief of pain from dental diseases.

**Design:**

This was a randomized, single-blind study.

**Setting:**

Hospital patients.

**Patients:**

Patients aged 16–60 years with acute pulpitis, acute apical periodontitis, or pericoronitis of the third molar of the mandible experiencing pain ≥3 on the 11-point numerical pain rating scale.

**Interventions:**

Three-minute pressure to sciatic nerves was applied by using the hands (hand pressure method) or by having the patients squat to force the thigh and shin as tightly as possible on the sandwiched sciatic nerve bundles (self-administered method).

**Outcomes:**

The primary efficacy variable was the mean difference in pain scores from the baseline.

**Results:**

One hundred seventy-two dental patients were randomized. The self-administered method produced significant relief from pain associated with dental diseases (*P* ≤ 0.001). The analgesic effect of the self-administered method was similar to that of the hand pressure method.

**Conclusions:**

The self-administered method is easy to learn and can be applied at any time for pain relief. We believe that patients will benefit from this method.

## Introduction

Stimulation of peripheral nerves increases the pain threshold 1–3. Theoretically, the larger peripheral nerve is stimulated, the stronger inhibition is. The sciatic nerve is the largest peripheral nerve, but pressure stimulation of the sciatic nerve is associated with hyperalgesia [4],[5]. For example, chronic pressure applied to the sciatic nerve, due to internal tension of the obturator muscle, or anatomical abnormalities in the piriformis muscle causes pain 4–6, and surgery to relieve the pressure results in immediate pain relief 7–9. We recently reported that acute pressure applied to the sciatic nerves can inhibit clinical pain, but not cold pressor pain 10–13. Although the period of relief is short term, the relief from pain is significant; and the method can be used repeatedly (the analgesic effect decreases if repeated continuously) 10–13.

It is unclear why the application of different types of pressure to the sciatic nerve has such strikingly different effects, with chronic pressure causing hyperalgesia and acute pressure providing significant pain relief. A hypothesis that may explain this phenomenon is that acid-sensitive ion channels (ASIC) are expressed in the neurons of the mammalian central and peripheral nervous systems. These ASICs have been proposed to be part of the mechanoreceptors and play an important role in the response to mechanical stimuli 14–17. So far, four ASIC proteins have been found to be expressed in the sciatic nerve [18].

Pain may occur at any time, such as at home, while traveling, during meetings, and at the farm, and no immediate method is available to relieve it. Herein, we present a novel self-administered analgesic method of an applying acute pressure block to the sciatic nerve and two clinical trials of the method. We believe that patients will benefit from this self-administered method.

## Methods

The two randomized trials were conducted in Department of Dentistry, Nanfang Hospital, Southern Medical University, China (the approval date: May 26, 2011). The open registration information of the trials of this study was ACTRN12611000747921. The pilot studies of the method were conducted in multiple centers (as indicated in the Acknowledgments section). All enrolled patients had pain from a dental disease (acute pulpitis, acute apical periodontitis, or pericoronitis of the third molar of the mandible) and were sequentially recruited by participating physicians using sealed envelope randomization method. None of the patients had previous experience with either method, and none used an analgesic medication in the previous 12 hours before participating in the experiment. The age range was 16–60 years, and all patients had an educational level higher than middle school. The allocation ratio is 1:1. Informed written consent was obtained, and subjects were instructed in use of the numerical rating scale (NRS), in which pain was scored from 0 (no pain) to 10 (maximum pain). Pain was assessed immediately before the intervention (baseline pain), and 1, 5, and 15 minutes after the intervention. The time of each intervention (self-administered pressure, hand pressure, or placebo) was 3 minutes.

The present experiments were conducted without following the minimum sample size because subjects are easy to collect due to the simple design of the study, short duration, and noninvasiveness of the intervention.

**Figure 1:**
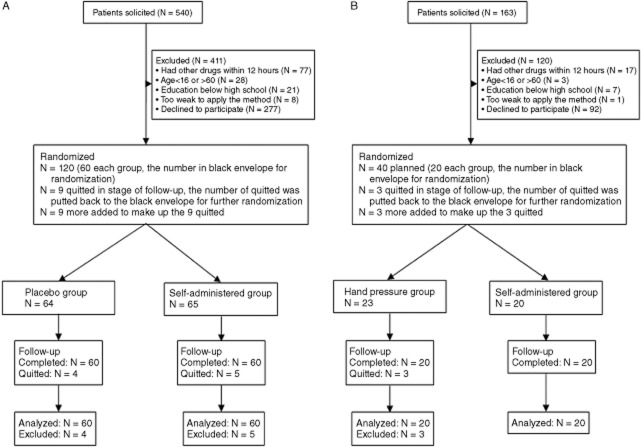
Participants flow through the study. (A) The first experiment examining the analgesic efficacy of the self-administered method vs the placebo intervention. (B) The second experiment comparing the analgesic efficacy of the self-administered method with the hand pressure method.

Among 703 dental patients screened for the two trials (Figure 1), 531 ineligible or declined to participate, and 172 eligible patents participated in the studies. First, randomized trial included in 129 patients, with nine who quitted during the experiments. Second randomized trial included in 43 patients, with three who quitted during the experiments. The first experiment examined the analgesic efficacy of the self-administered method compared with the placebo treatment, and the second compared the analgesic efficacy of the self-administered method with the hand pressure method.

**Figure 2:**
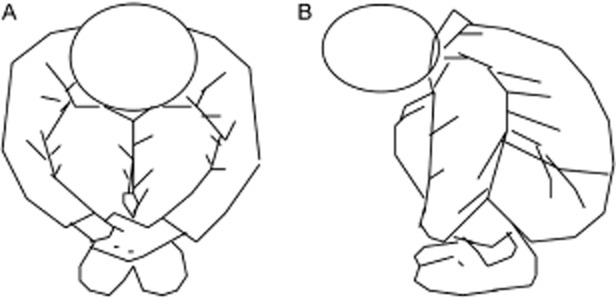
The self-administered method, or tight squatting down method. (A) Front view, (B) side view.

The self-administered method: Figure 2A (front view) and Figure 2B (side view) show a subject squatting down to force the thigh and shin to stretch and press as tightly as possible on the sandwiched sciatic nerve bundles, with the hips close to the heels and the arms hugging the ankles tightly. The squatting pressure lasted for 3 minutes.

The placebo intervention for the study of the self-administered method: Patients sat on a soft chair, with their arms surrounding their knees and their head and neck inclined slightly forward.

**The hand pressure method**: A pressure of 10–20 kg per hand was applied to the sciatic nerve at the backs of both thighs for 3 minutes while the patients were in the prone position, according to the reports by He et al. 10–13.

### Statistical Analysis

The baseline scores, sex, and age of the participants were compared between the self-administered method and placebo groups, or the self-administered method and hand pressure method groups by using the χ^2^ test and *t*-test. The NRS were assessed within groups or between groups using Mann–Whitney *U*-test. All tests were two sided, and a *P* value of <0.05 was considered to indicate significance. The analysis included the per-protocol data. All statistical analyses were performed by using SPSS statistical software (release 13.0, Chicago, IL, USA).

## Results

**Figure 3:**
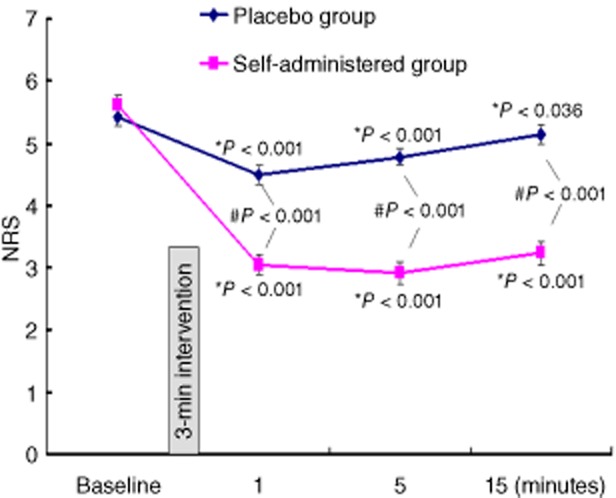
The analgesic effect of the self-administered method. Data are presented as mean numerical rating scale (NRS) score ± standard error of the mean (SEM). *P value indicates the significance of changes from the baseline for both self-administered or placebo group. #*P* value indicates the significant difference in pain relief between the self-administered and placebo groups.

Table 1 displays the demographic data for the patients. The self-administered method provided significantly greater relief of pain than the placebo method (^#^*P* < 0.001 for all three time points between the two groups, Figure 3). Of the 60 patients who practiced the self-administered method, 46 experienced pain relief (relief was defined as a decrease ≥2 in NRS score). The average pain relief was significant at all three time points after acute pressure block of the sciatic nerve (*P < 0.001) (Figure 3). In contrast, the placebo method provided a much less relief, and the pain returned quickly (Figure 3). In the placebo group, 9 out of 60 patients of the placebo group felt relief after the placebo intervention; of these nine patients, five experienced return of pain at 15 minutes.

**Table 1:** Demographic data of patients in experiment 1

	Self-Administered Group	Placebo Group	*P* Value
Participants	60	60	—
Male (%)	51.67%	45.00%	0.469
Age	31.49 (±9.49)	32.33 (±9.37)	0.672
Baseline NRS	5.62 (±1.14)	5.43 (±1.12)	0.917
Weight (kg)	56.89 (±10.14)	55.23 (±9.69)	0.464
Height (cm)	162.63 (±8.74)	163.92 (±8.90)	0.523

Age, gender, baseline numerical rating scale (NRS), weight, and height: mean (±standard deviation [SD]), *t*-test.

**Figure 4:**
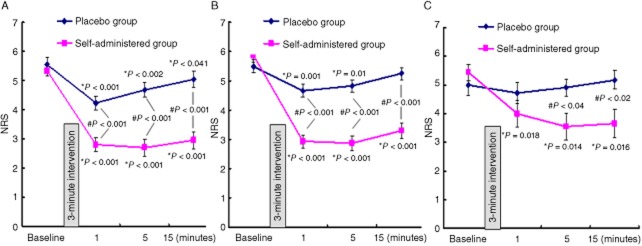
The analgesic effect of the self-administered method on different patient subgroups. (A) acute pulpitis, (B) acute apical periodontitis, and (C) pericoronitis of the third molar of the mandible. Data are presented as mean numerical rating scale (NRS) score ± standard error of the mean (SEM). *P value indicates the significance of changes from the baseline for both self-administered or placebo group. #*P* value indicates the significant difference in pain relief between the self-administered and placebo groups.

We further analyzed the analgesic efficacy of the self-administered method on different patient subgroups. The demographic characteristics of the patient subgroups (acute pulpitis, acute apical periodontitis, or pericoronitis of the third molar of the mandible) in experiment 1 are shown in Table 2. The self-administered method provided significant pain relief for acute pulpitis (Figure 4A, *P < 0.001 for the three time points), acute apical periodontitis (Figure 4B, *P < 0.001 for the three time points), and pericoronitis of the third molar of the mandible (Figure 4C, *P = 0.018, 0.014, and 0.016 at 1, 5, and 15 minutes after the intervention, respectively).

**Table 2:** Demographic data of patients sub-group of acute pulpitis, apicitis, and periodontitis in experiment 1

	Self-Administered Group	Placebo Group	*P* Value
Acute pulpitis			
Participants	32	25	—
Male (%)	53.13%	48.00%	0.707
Age	30.71 (±8.37)	31.44 (±9.12)	0.796
Baseline NRS	5.87 (±1.33)	5.50 (±1.06)	0.290
Weight(kg)	54.76 (±10.49)	54.53 (±9.98)	0.950
Height (cm)	160.65 (±8.49)	164.73 (±10.07)	0.223
Apicitis			
Participants	19	24	—
Male (%)	42.10%	54.17%	0.444
Age	33.88 (11.95)	35.63 (±9.69)	0.634
Baseline NRS	5.33 (±0.86)	5.56 (±1.16)	0.244
Weight (kg)	55.86 (±9.66)	57.75 (±9.55)	0.594
Height (cm)	161.86 (±8.62)	164.44 (±8.15)	0.407
Periodontitis			
Participants	9	11	—
Male (%)	66.67%	18.19%	0.027
Age	28.75 (±6.04)	26.50 (±6.21)	0.475
Baseline NRS	5.44 (±0.88)	5.00 (±1.18)	0.485
Weight (kg)	64.14 (±7.90)	51.50 (±9.17)	0.014
Height (cm)	169.00 (±7.62)	161.38 (±8.68)	0.096

Age, gender, baseline numerical rating scale (NRS), weight, and height: mean (±standard deviation [SD]), *t*-test.

The self-administered method provided greater pain relief compared with the placebo method for all three types of disease. The difference between acute pressure relief and the placebo relief was significant: ^#^*P* < 0.001 for all the three time points after intervention for pulpitis and acute apical periodontitis, and ^#^*P* = 0.04 and 0.02 at 5 and 15 minutes, respectively, after intervention for pericoronitis.

**Figure 5:**
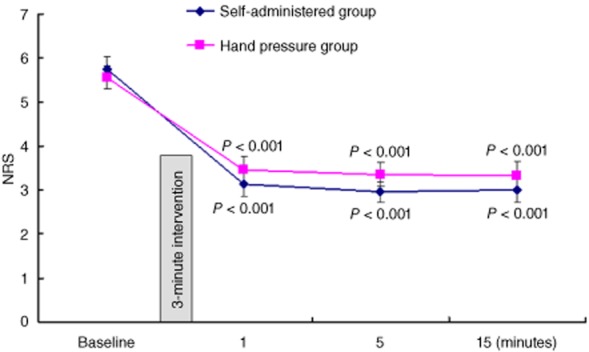
Comparison of the analgesic effect of the self-administered method and the hand pressure method. Data are presented as mean numerical rating scale (NRS) score ± standard error of the mean (SEM). *P value indicates the significance of changes from the baseline for both self-administered or placebo groups.

In our previous reports that utilized the hand pressure method, pressure applied to the sciatic nerve at the back of both thighs provided significantly greater relief of pain compared with same amount of pressure applied to parallel regions on the front of the thighs. Effective pressure on any accessible area along the sciatic nerve provides pain relief, and the relief is reduced if the pressure is applied at a distance from the sciatic nerve 11–13,19. Herein, we compared the analgesic efficacy of the self-administered method with the hand pressure method. Table 3 displays the demographic data for the patients in experiment 2. The squatting down method produced a similar relief of pain as, or even slightly better relief than, the hand pressure method did (Figure 5).

**Table 3:** Demographic data of patients in experiment 2

	Self-Administered Group	Hand Pressure Group	*P* Value
Participants	20	20	—
Male (%)	65.0%	55.0%	0.531
Age	31.60 (±11.26)	33.30 (±9.23)	0.605
Baseline NRS	5.75 (±1.21)	5.55 (±1.15)	0.594
Weight (kg)	58.15 (±9.76)	54.64 (±9.24)	0.524
Height (cm)	161.68 (±9.15)	161.35 (±9.13)	0.486

Age, gender, baseline numerical rating scale (NRS), weight, and height: mean (±standard deviation [SD]), *t*-test.

## Discussion

Stimulation of peripheral nerves elevates the pain threshold. Thus, for the purpose of inhibition of pain, stimulation of the sciatic nerve is a conceivable idea, given the large size of the sciatic nerve. We introduce a simple self-administered method to relieve dental pain. With a right squatting down as introduced, the entire sciatic nerve receives pressure; pain was relieved immediately and significant.

After our method spread out from the study centers, several doctors even contacted us about learning from their patients, that pain occasionally disappeared when their patients squatted down for a few min. We reasoned that the relief was due to an acute pressure blockade of the sciatic nerve, similar to what we recorded in this study. In contrast, a tingling sensation or intolerable pain often occurs when a subject sits in a cross-legged position for an extended period. This observation may be similar to the hyperalgesia caused by chronic pressure on the sciatic nerve.

Spinal dorsal horn the wide dynamic neurons (WDR) are the first synaptic relay point for afferent pathways, and they play an important role in modifying the transmission of noxious input [20],[21]. According to the gate control theory of pain [22], stimulation of large-diameter afferent fibers inhibits second-order neurons in the dorsal horn and prevents impulses carried by small-diameter fibers from being transmitted further; the resulting analgesic effect is considered to be short lived, occurring rapidly, and is thought to involve WDR neurons 22–24. We recently demonstrated that pressure applied to the rat sciatic nerve caused immediate inhibition of WDR neurons [25]. These data may partially explain the immediate analgesic effect of the acute pressure block of the sciatic nerve.

Interestingly, acupressure, a form of acupuncture, applies pressure to the Yao Yang Guan acupuncture point to relieve acute sciatica pain and low back pain [26],[27]. This acupuncture point is below the spinous process of the fourth lumbar vertebrae, where the sciatic nerve branches out from the spinal cord. The analgesic mechanism of acupressure is largely unknown. According to our study, effective pressure on any accessible area along the sciatic nerve will provide rapid pain relief, and the effectiveness is reduced if the pressure is applied at a distance from the sciatic nerve. Thus, future studies that identify whether the relief from acute sciatica pain or low back pain by the acupressure is due to the acupressure stimulation of the acupuncture point, or to the acute pressure stimulation of the sciatic nerve may provide insight into several traditional Chinese medicine techniques.

For the hand pressure method, a pressure of 10–20 kg/hand is required to provide pain relief 11–13. The self-administered method involves squatting down; therefore, the pressure to the sciatic nerve, per unit area, should be much smaller than the 10–20 kg/hand pressure administered by the hand. However, in the self- administered method, the entire sciatic nerve receives pressure; therefore, the total pressure to the sciatic nerve should be greater than the 10–20 kg, which results from the hand pressure method. Thus, the extent of the nerve area that receives pressure appears to be an important factor in pain relief for this method.

We used a 3 minutes of intervention for the self-administered pressure method, instead of 2 minutes used in He et al.'s studies, because our pilot studies shows that some patients need more time for pain relief. Usually, a sample size of 20 subjects was enough to reach significance in tests of dental diseases. However, the present experiments were conducted without following the minimum sample size because subjects are easy to collect due to the simple design of the study, short duration, and noninvasiveness of the intervention.

The first limitation of this study was the small sample size, which limited the subgroup analysis. The second limitation is that sitting on a chair may be not a good control for the self-administered method, even though the positions of the patients for the control method is similar to that for the self-administered method, with patients' arms hugging the legs. The third limitation was the single-blind design. We did not conduct a double-blinded study because the method appeared to be highly effective for reducing pain; thus, the doctors were easily able to identify the placebo or the treatment group during the experiment. However, a double-blind study could demonstrate the efficacy of the treatment and could provide results that are more precise. Thus, a double-blind study would be a help to confirm our results.
